# An AI-Driven Multimodal Monitoring System for Early Mastitis Indicators in Italian Mediterranean Buffalo

**DOI:** 10.3390/s25154865

**Published:** 2025-08-07

**Authors:** Maria Teresa Verde, Mattia Fonisto, Flora Amato, Annalisa Liccardo, Roberta Matera, Gianluca Neglia, Francesco Bonavolontà

**Affiliations:** 1 Department of Veterinary Medicine and Animal Production, University of Naples Federico II, 80137 Naples, Italy; mariateresa.verde@unina.it (M.T.V.); roberta.matera@unina.it (R.M.); gianluca.neglia@unina.it (G.N.); 2 Department of Electrical Engineering and Information Technology, University of Naples Federico II, 80125 Naples, Italy; mattia.fonisto@unina.it (M.F.); flora.amato@unina.it (F.A.); annalisa.liccardo@unina.it (A.L.)

**Keywords:** infrared thermography, udder health, machine learning, instrument and measurements, artificial intelligence (AI), early disease detection

## Abstract

**Highlights:**

**What are the main findings?**

- An AI-driven thermal imaging system, synchronized with robotic milking, enables continuous, non-invasive monitoring of udder health in Italian Mediterranean buffalo.
- A SegFormer-based neural network accurately segments the udder and extracts the maximum skin temperature, showing a significant correlation with somatic cell count (SCC).

**What are the implications of the main findings?**

- The system allows for the early detection of subclinical mastitis, enabling timely veterinary intervention before clinical signs appear.
- This method supports precision livestock farming by reducing stress, avoiding unnecessary antibiotic use, and improving milk quality and farm sustainability.

**Abstract:**

Mastitis is a significant challenge in the buffalo industry, affecting both milk production and animal health and resulting in economic losses. This study presents the first fully automated AI-driven thermal imaging system integrated with robotic milking, specifically developed for the real-time, non-invasive monitoring of udder health in Italian Mediterranean buffalo. Unlike traditional approaches, the system leverages the synchronized acquisition of thermal images during milking and compensates for environmental variables through a calibrated weather station. A transformer-based neural network (SegFormer) segments the udder area, enabling the extraction of maximum udder skin surface temperature (USST), which is significantly correlated with somatic cell count (SCC). Initial trials demonstrate the feasibility of this approach in operational farm environments, paving the way for scalable, precision diagnostics of subclinical mastitis. This work represents a critical step toward intelligent, automated systems for early detection and intervention, improving animal welfare and reducing antibiotic use.

## 1. Introduction

Mastitis is one of the major health issues in buffalo farming, significantly impacting both animal health and farm profitability. This condition is an inflammation of the mammary gland that can occur in two main forms: clinical and subclinical. When the infection occurs, neutrophil cells migrate rapidly from the blood to mammary tissue to counteract the pathogens responsible for the inflammation and can also be found in the milk. This process leads to an increase in somatic cell count (SCC) in milk, with neutrophils comprising a substantial proportion of these cells. Elevated SCC is a primary indicator of mammary infection and is commonly used to diagnose mastitis [1]. Clinical mastitis shows visible signs, such as oedema, pain, and physical and chemical changes in the milk [2]. In contrast, when subclinical mastitis occurs, no visible symptoms are recorded, with the exception of an increase in SCC, complicating an early diagnosis [3]. Even though buffalo are traditionally considered less susceptible to mastitis compared to cows, some studies have reported a higher prevalence of subclinical mastitis in this species [4] compared to dairy cattle. Despite being asymptomatic, this type of mastitis negatively affects milk quality, resulting in an increase in somatic cell count, a reduction in lactose, fat, protein, and, obviously, milk yield. Furthermore, if left undiagnosed and untreated, subclinical mastitis can progress to chronic or clinical forms, causing more serious consequences for animal health and farm productivity [5]. Traditional methods for diagnosing subclinical mastitis, such as SCC [4] and the California mastitis test (CMT) [6], are widely used but limited in sensitivity and timeliness. These tests often require repeated milk sampling and laboratory analysis, which can be costly.

In this context, infrared thermography presents a promising diagnostic solution. Udder surfaces affected by mastitis exhibit increased temperatures due to the inflammatory response and increased blood flow. Infrared technology detects thermal radiation emitted by mammary tissues, allowing even minor temperature variations linked to inflammation to be identified. This facilitates early disease diagnosis, detecting thermal anomalies before more evident symptoms appear [7]. Numerous studies confirm the effectiveness of thermography for mastitis detection, such as [8]. The main advantage of infrared thermography is that it is a contactless technology, unlike other diagnostic methods [9]. This allows animal health monitoring without frequently handling the udder (e.g., during veterinary examinations confirming pathology), thereby reducing animal stress or taking milk samples to perform costly microbiological analyses. For these reasons, this method is faster and less invasive than commonly adopted techniques. Rapid diagnosis is essential to allow prompt treatment, prevent milk composition alterations, and preserve farm profitability. Mastitis increases SCC in milk while reducing protein and fat concentrations, factors that compromise milk’s commercial quality and, in severe cases, can prevent it from being sold due to infection. Despite its advantages, infrared thermography presents challenges for on-farm applications. Environmental conditions, such as temperature, humidity, and ventilation, vary widely on farms and can affect measurement accuracy, complicating the detection of small but significant temperature changes. Additionally, continuous animal movement makes capturing stable, comparable thermal images challenging. Effective thermal imaging requires skilled personnel to operate the camera and interpret the results accurately. In many cases, integrating thermographic data with other clinical or diagnostic information (such as production, SCC, and electrical conductivity) is necessary to avoid false positives or negatives. Continuous temperature monitoring through thermography is only feasible if performed consistently, but in practice, this requirement can be difficult to meet, particularly on a large scale. Implementing thermography into daily routines may necessitate operational changes and additional time for farm management, and these adjustments become increasingly complex as farm size grows.

An additional obstacle to continuous thermographic monitoring is the lack of “standardization” of the udder, which can vary significantly from one animal to another in the Italian Mediterranean Buffalo in terms of shape, symmetry, udder attachment, teat position and length, height from the ground, and other significant characteristics. However, the thermal image captured by the infrared camera not only includes the udder and the target, but also other areas close to it, such as the abdomen, the inner thigh, and elements of the milking robot. Therefore, if the udder is not correctly identified, the recorded temperatures may not match those of the udder, leading to inaccurate measurements and errors in the diagnosis of mastitis. All these issues can be addressed by developing an appropriate automatic thermal image capture system, enhanced by artificial intelligence (AI) algorithms capable of accurately identifying the udder. This approach aims to achieve precise automatic segmentation of the buffalo udder, thereby solving the abovementioned problems and improving the accuracy of buffalo mastitis detection. Moreover, it can process and standardize images, ensuring uniformity through adjustments such as colour normalization and temperature scale calibration, thus facilitating reliable comparisons and subsequent analyses. This integrated approach not only improves diagnostic reliability but also supports continuous and non-invasive monitoring, which is crucial for managing animal health on a large scale. The adoption of these technologies marks a significant step forward in achieving precise and efficient livestock health management. Therefore, the main objective of this study is to establish and refine an effective methodology based on artificial intelligence that enables the use of infrared thermography as a monitoring tool for subclinical mastitis in Italian Mediterranean buffalo, even on farms with many animals. Specifically, the proposed methodology is based on developing and employing an automated system for capturing and analyzing thermal images of the udder, with the thermal camera strategically placed at the side and rear of the milking robot.

The integration and synchronization of thermographic detection with automated milking, which is already unsupervised, allows the following benefits:

1. controlling geometric parameters that can influence measurement, ensuring thermal images are consistently taken at the same distance and angle relative to the udder, with animals remaining nearly stationary in the robot for the entire duration of milking;
2. compensating for the effects of ambient temperature and humidity on measurement using appropriate sensors, allowing the analysis to consider only the temporal dynamics of influencing factors, as measurements are always conducted in the same location within the barn;
3. achieving continuous, constant monitoring of the udder health of each animal without adding extra tasks to the daily routine, minimizing stress for the animals;
4. producing synchronized thermal images that can be integrated with other heterogeneous data already collected by the milking robot (e.g., quarter production, electrical conductivity, and somatic cell count) to enhance the robustness and reliability of predictive models.

The monitoring system is enhanced with appropriately trained segmentation neural networks that, once a thermal image is acquired, perform the following steps:

1. identification and segmentation of the udder in the image;
2. automatic extraction of the maximum udder skin surface temperature from the segmented region;
3. preliminary evaluation of the correlation between the maximum temperature and somatic cell count values to generate udder health indicators.

It is important to note that, at present, the system does not include an integrated diagnostic model for the automated detection of subclinical mastitis. Instead, the current setup supports veterinarians by providing real-time udder health information, with the observed temperature–SCC correlation (validated via *t*-tests) laying the groundwork for future diagnostic tools.

Initial results obtained from the creation and field application of this first prototype of a fully automated mastitis diagnostic system based on infrared thermography monitoring confirm the potential of the proposed method.

In summary, the core contribution of this study lies in the development of a fully automated, AI-driven thermographic monitoring system integrated with a robotic milking unit. Central to this approach is a SegFormer-based neural network trained to segment the buffalo udder in thermal images and extract the maximum skin temperature, which correlates with the somatic cell count. The following sections describe the design of the thermal acquisition system, the training of the segmentation model, and its integration into a real-time monitoring framework.

The following sections provide detailed descriptions of the design and development of the automated thermal image acquisition system and the procedures implemented to label thermographic data and synchronize them with the heterogeneous data collected by the robot (Section 2), the pre-processing of the dataset and the training of the SegFormer transformer-based neural network for the segmentation and extraction of relevant thermographic information accounting for meteorological factors (Section 3), preliminary results demonstrating the potential of the proposed methodology (Section 4), and conclusions and potential future developments (Section 5).

## 2. Design and Development of the Automatic Thermal Imaging System Synchronized with the Milking Routine

**Figure 1:**
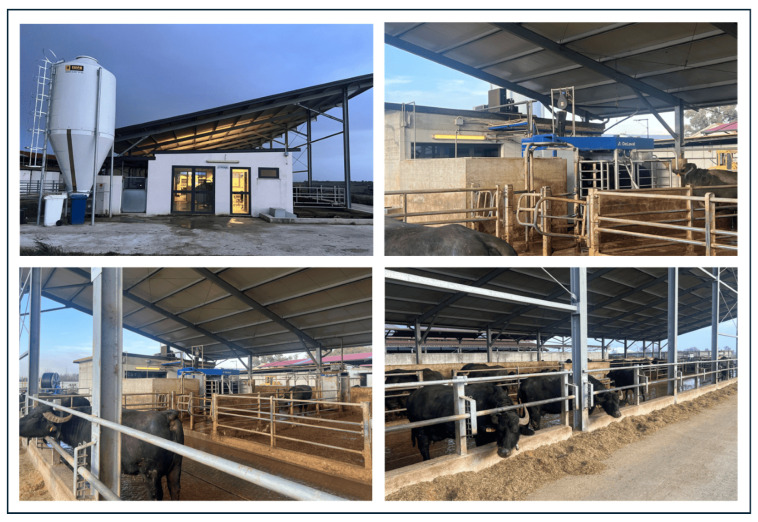
Pictures from the buffalo farm, equipped with a milking robot, in Cancello ed Arnone.

The primary objective of the research activities was to develop an automated system for capturing thermal images of the udder, synchronized with the milking phase and capable of handling a substantial amount of data. Synchronizing the acquisition of udder thermal images with the milking phase allows for easy association of each image with the parameters detected by the robot, which are typically used to assess the health status of the buffalo. This facilitates the subsequent analysis phase and the creation of predictive models. Moreover, the automated image acquisition enables detection directly in the barn under a semi-forced circulation system, where the buffalo autonomously decide when to be milked based on their own needs, thus avoiding additional stress for the animals (Figure 1).

**Figure 2:**
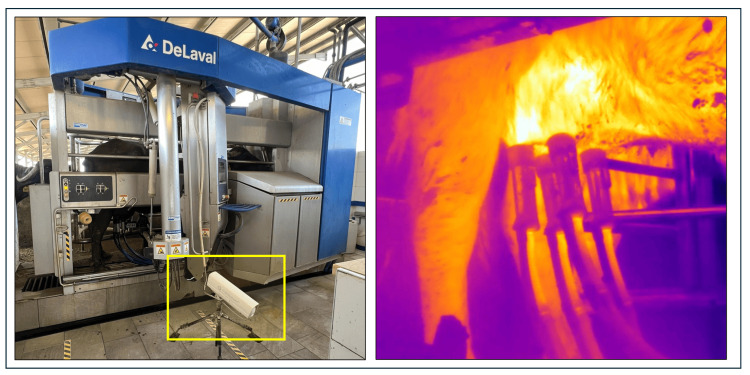
Installation of the thermal camera in the milking robot and example of acquired infrared image.

In fact, without the implemented automated acquisition system, in this type of innovative barn, it would have been necessary to use multiple operators to manually capture thermal images, ensuring continuous coverage throughout the 24-h period during which buffalo may be milked. This would prevent data loss and allow for uninterrupted monitoring of all animals. The use of multiple operators would also introduce additional factors of variability and uncertainty into the measurement system, including fatigue effects that arise from prolonged repetitive tasks. To illustrate, in a single day of acquisition, with a group of 60 buffalo milked approximately 2.4 times per day, the images to be processed and correlated with other data amount to 144. The thermal camera has been strategically positioned on the lateral and rear sections of the milking robot (Figure 2).

The current configuration, with the infrared camera positioned at the rear of the milking robot, only captures one side of the udder. This monolateral view may limit the detection of localized thermal anomalies, particularly in the anterior quarters or the contralateral side. Furthermore, the notable morphological variability of the udder in Italian Mediterranean buffaloes, especially in terms of symmetry, teat placement, and attachment, can affect segmentation accuracy and thermal data reliability.

**Figure 3:**
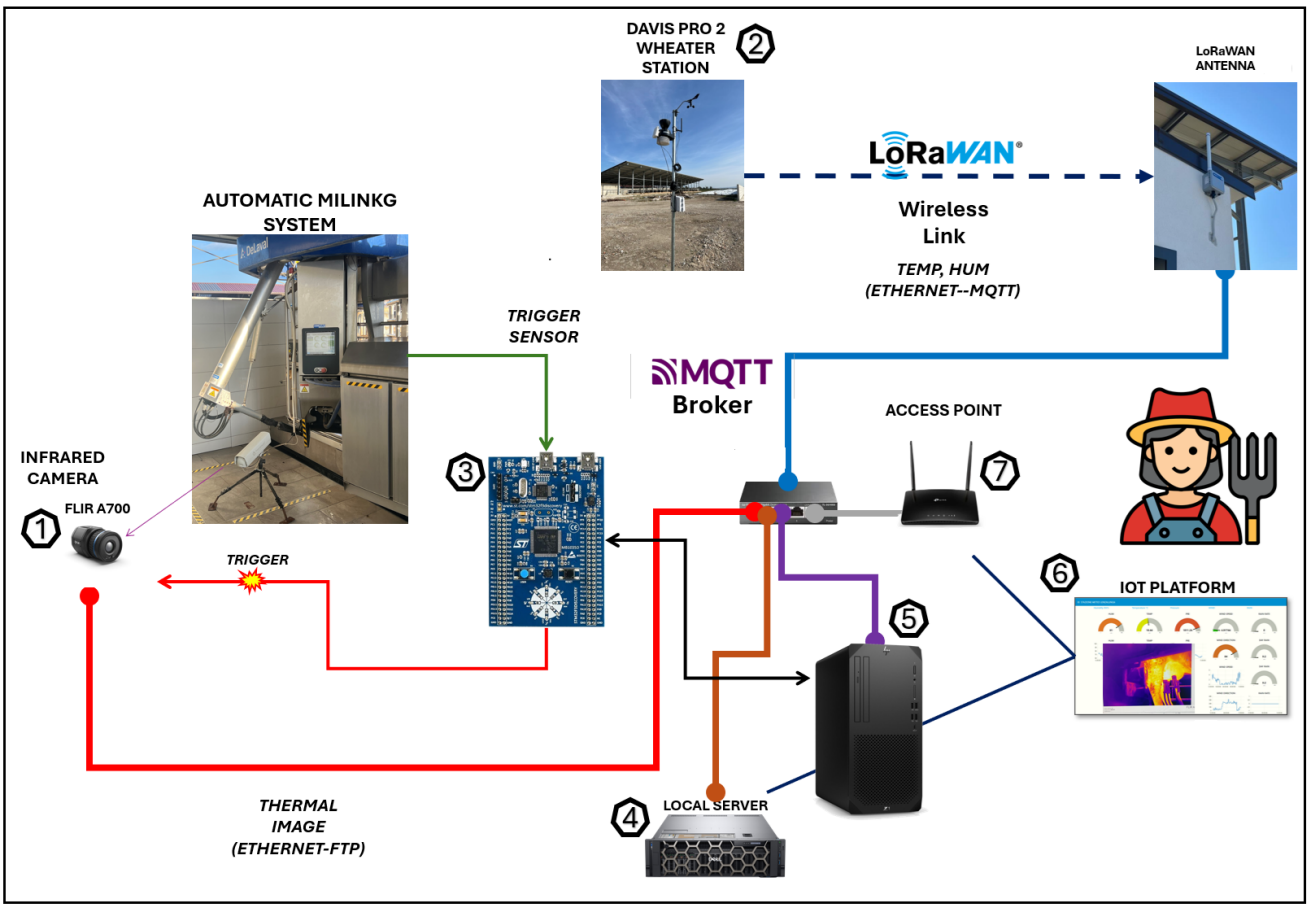
Automatic measurement station setup.

However, although installing an infrared camera on the milking robot appears to be the most logical choice, considerable technical effort was required to design and implement a custom system capable of ensuring synchronized acquisition and automatic, reliable storage of thermal images of the udders, given the lack of standardized protocols. The automated measurement station developed, shown in Figure 3, consists of the following:

1. a FLIR A700 infrared thermal camera positioned on the rear of the robot at an angle that optimally centers the udder within the camera’s field of view;
2. a weather station to capture ambient temperature and humidity data, allowing for compensation in udder temperature measurements;
3. a control logic unit responsible for generating a timely trigger signal for the thermal camera, ensuring a new thermal image is captured at the start of each milking phase;
4. a local server for storing all heterogeneous data produced by the various sensors;
5. a workstation running a custom-developed software application that temporally aligns the data acquired from the additional sensors (thermal images and meteorological data) with the data already produced by the robot for each milking (e.g., somatic cell count, electrical conductivity, and production) by matching the timestamps from both sources and extract udder maximum temperatures using the trained segmentation network while accounting for meteorological factors;
6. an IoT platform that graphically displays time-series variations in relevant monitored system variables, as well as real-time alerts and notifications of anomalies, such as when maximum udder skin surface temperature (USST) exceeds a set threshold;
7. an access point that allows remote user access to data via a SIM card, even in rural areas without wired Internet access, while ensuring data security by filtering access to unauthorized users.

### 2.1. Thermal Camera

**Figure 4:**
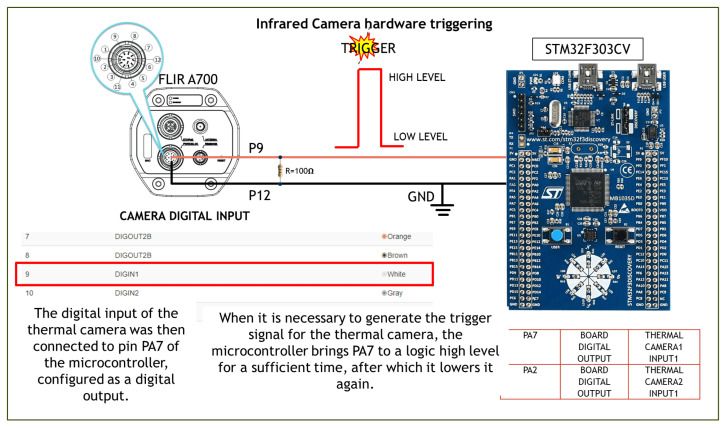
Thermal camera control device.

The chosen thermal camera sensor is the FLIR A700, which offers high-resolution thermal imaging (640 × 480 pixels) with a sensitivity of <30 mK, supports temperature measurements from −20 °C to 2000 °C and various lens options (14°, 24°, and 42°), and integrates seamlessly into industrial systems with advanced connectivity protocols. The thermal camera is enclosed in a waterproof IP66 housing with a germanium lens in order to protect it from potential impacts with the milking equipment, animal waste (urine and feces), as well as water and detergent used by the robot during cleaning cycles. The lens blocks wavelengths in the UV (ultraviolet) and VIS (visible) spectra but allows IR (infrared) wavelengths starting from 2 μm to pass through. In addition to its very good performance characteristics, the thermal camera features digital I/O (input/output) ports on the rear panel, facilitating seamless integration into an automated measurement and control system. For example, the digital inputs can be configured to detect a transition from low to high in a direct current voltage signal (trigger event) and perform a specific action upon the trigger event, such as capturing a thermal snapshot or recording a video clip of several seconds (Figure 4). It is possible to capture a thermal image at a given time instant by timing a trigger signal. A digital output can be configured to generate an alarm signal or activate a relay when a set temperature threshold is exceeded. The thermal camera is installed on the local network with the IP address 192.XXX.X.151.

Once captured, the thermal image is transferred from the thermal camera to the local server via FTP over Ethernet to the local address IP 192.XXX.X.179.

### 2.2. Weather Station

Davis Pro 2 was selected as a weather station, equipped with (i) a high-precision temperature sensor with a resolution equal to 0.1 °C and accuracy equal to ±0.3 °C, and (ii) a humidity sensor with a resolution of 1% and accuracy of 3% within the range 4–90%. In addition to temperature and humidity data, which compensated for the temperature measurements taken with the infrared thermal camera, the weather station provided wind speed/direction and atmospheric pressure values, useful for estimating additional parameters such as methane and ammonia gas emissions. The weather station was installed on a pole 3 m above ground near the experimental barn. A small solar panel and a lead-acid battery ensured its operation without the need for a network cable power supply. The atmospheric data collected were transmitted via a wireless network.

It was, therefore, necessary to establish a wireless link between the weather station (sensor node) and the automated data collection system. LoRaWAN was chosen as the communication protocol due to the intrinsic characteristics that make it suitable for rural areas, i.e., (i) a transmission range up to 15 km in the open field, (ii) low power dissipation, and (iii) high solution scalability.

A LoRaWAN gateway, installed near the milking robot, ensured coverage across the entire facility. The weather station, equipped with a LoRaWAN interface, served as a sensor node within the star architecture network established by the gateway. Approximately every 20 min, the weather station transmitted the updated ambient temperature and humidity data to the gateway. This data rate represents an optimal trade-off between the variability dynamics of environmental parameters and the power consumption of the weather station; it ensures the operation of the weather station even during night-time hours and on cloudy days when the battery cannot be recharged by the panel due to a lack of sunlight.

The gateway was connected to a network switch via an Ethernet cable, had its own local IP address (IP address 192.XXX.X.150), and published the temperature and humidity data to an external proprietary MQTT broker provided by the University of Naples Federico II, at IP address 143.XXX.XX.193:21883 [10].

The MQTT Client running on the Workstation subscribed to the MQTT topic carrying the temperature and humidity data, receiving and storing the data on the local server.

### 2.3. Control System

The control logic unit was responsible for generating the trigger signal for the thermal camera to take a thermal snapshot each time a new milking phase was about to begin. The core of the control logic unit was an ST32F303VC microcontroller with an ARM architecture.

**Figure 5:**
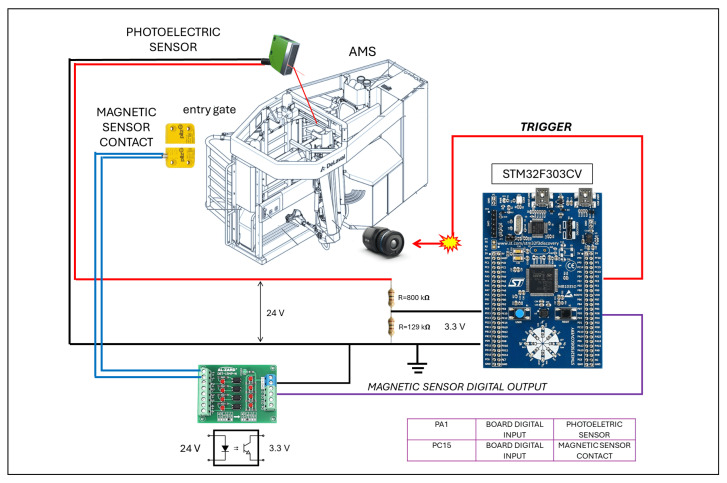
General synchronization system.

To detect when a buffalo entered the robot and a new milking phase began, the microcontroller monitored the status of two 0/1 digital output sensors: (i) a photocell and (ii) a non-contact magnetic sensor (Figure 5).

The photocell operated in reflection mode, was installed at the top center of the milking robot, and pointed downwards. When the robot was empty, the photocell outputted zero voltage (low logic level). When a buffalo began to enter the robot, the light beam emitted by the photocell was reflected off the buffalo’s body, and the digital output switched from low to high, signaling that a new animal had entered the robot.

However, the photocell alone could not confirm that a buffalo had fully entered the robot or that milking was ready to begin. Often, especially when a buffalo encountered the robot for the first time and was unfamiliar with the process, it was hesitant. The buffalo would enter, go halfway, triggering the photocell (indicating presence), then stop, survey its surroundings and exit, resetting the photocell to low (indicating absence), and so on.

This uncertain behavior was repeated, with the buffalo moving forward and back several times before fully entering the robot and assuming the correct milking position. As a result, a series of state changes were recorded on the photocell output. In practice, the buffalo was not a conveyor belt object, so the time it took to fully enter the robot varied and was unpredictable.

As a result, the photocell output alone could not trigger the camera event, as it did not reliably indicate the correct position of the buffalo; even with an additional delay, a usable thermal image of the udder could not be captured. After the buffalo entered the robot, the entry gate attempted an initial closure to secure it inside. If the buffalo had fully entered, the rear gate closed without a problem. Otherwise, it reopened.

Further closing attempts were made until the buffalo was fully inside the robot. A few seconds after the entrance gate closed, the robot arm began to move to attach the teat cups one by one, initiating the milking phase.

A non-contact magnetic sensor on the entrance gate signaled whether the gate was open (sensor output at high logic level) or closed (sensor output at low logic level).

**Figure 6:**
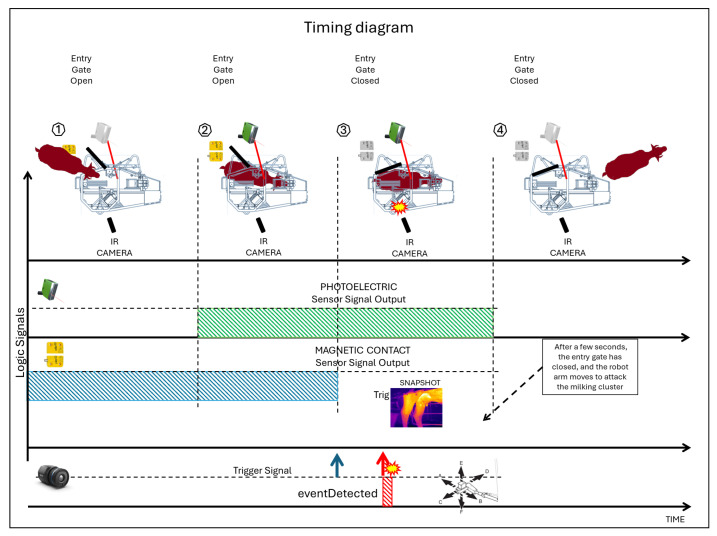
Diagram for the correct timing in infrared image capturing.

By monitoring the status of the non-contact magnetic sensor in addition to the photocell, the control unit can recognize that the buffalo has entered the robot, is in the correct position, and that the start of milking is imminent. In the short time between the closing of the entry gate and the start of the movement of the robot arm, the control unit must generate the trigger signal for the thermal camera to take the optimum thermal image of the udder (Figure 6).

**Figure 7:**
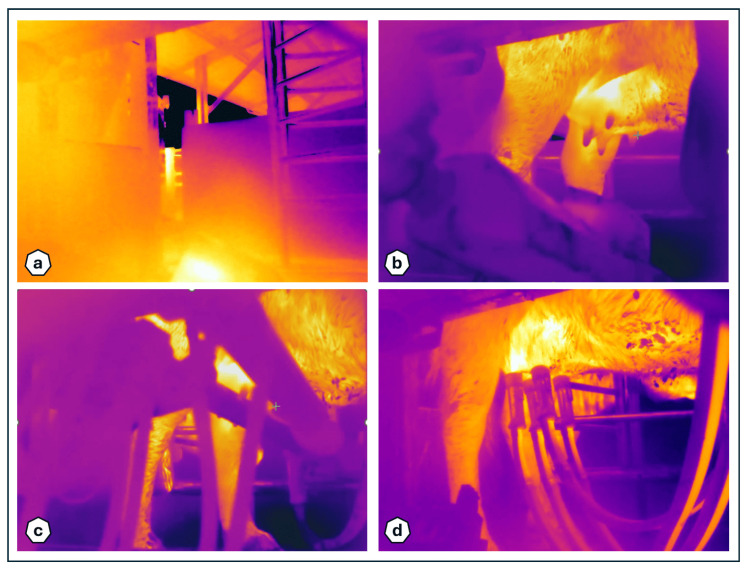
Examples of acquired infrared images: (**a**) anticipated capturing, (**b**) perfect timing, (**c**,**d**) delayed capturing.

In fact, if the shot is taken early, as mentioned above, there is no guarantee that the udder is correctly framed, while an excessive delay would compromise image acquisition as the camera’s view would then be obstructed by the moving robotic arm (Figure 7).

#### Microcontroller Firmware

**Figure 8:**
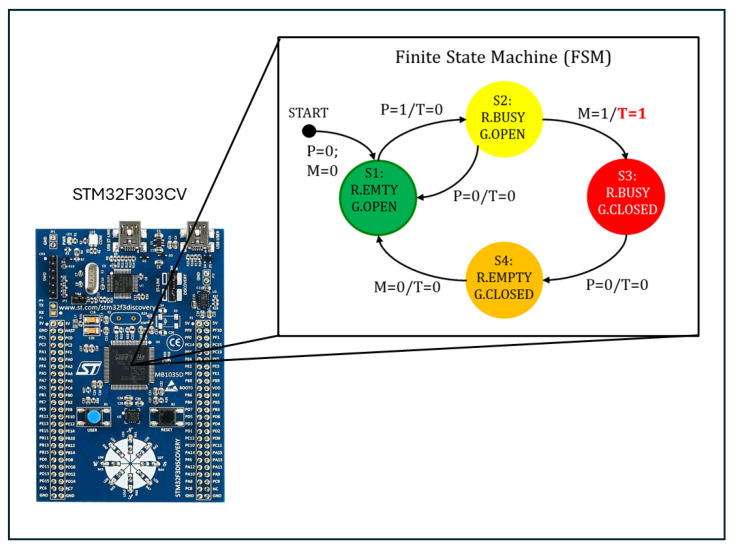
Finite state machine for system control.

The firmware of the microcontroller for the optimal management of the thermal photocell trigger was structured according to the architecture of a finite-state machine, consisting of four states with two inputs: the photoelectric sensor (P) and the contactless magnetic sensor (M) and a single output, the trigger (T) (Figure 8).

At start-up, the finite state machine enters the initial state S1, indicating that the robot is empty and the door is open. Switching the photocell output P from 0 to 1 causes the transition to state S2. In this state, the robot is occupied, and the access door is still open. It is possible to return from state S2 by switching the photocell output from 1 to 0 or to go to state S3 by closing the entrance gate (M = 1). The transition from state S2 to state S3 (robot occupied and entrance gate closed) causes the output T = 1 to change, i.e., trigger generation for the camera. At the end of the milking phase, the buffalo leaves the robot, and the output of the photocell (P) changes from 1 to 0, determining the transition of the finite state machine from state S3 to state S4 (robot empty and entrance gate closed). Only when the robot opens the entrance gate to allow a new buffalo to enter does the output of the contactless magnetic sensor switch from 1 to 0 and return to the initial state S1.

### 2.4. Local Storage Server

The local server is a Dell PowerEdge R550 Tag F9BTYP3 with two hard drives, one 1TB SSD and one 4TB SSD, 32GB RAM, and a 16-core 3.90GHz CPU. Each time the camera captures a thermal image, it is transferred to the local server via FTP protocol. Since a thermal image is approximately 640 KB in size, the local server can host millions of thermal images, providing continuous monitoring and data history of all animals in the barn for several years. The local server also historizes the weather data collected by the weather station sent by the workstation and prompts it for image analysis whenever a new thermal image is available.

### 2.5. High-Performance Workstation for Image Analysis

The workstation is a high-performance machine consisting of (i) an AMD Ryzen Threadripper PRO 5975WX processor running at 3.6 GHz, (ii) 4x Nvidia Quadro RTX A6000 GPUs with 48 GB GDDR6 VRAM, and (iii) 256 GB of DDR5 RAM running at 6000 MHz. The MQTT client is also running on the workstation, which has subscribed to the weather data topic produced by the LoRaWAN [11] weather station; therefore, every 20 min, the workstation receives updated temperature and humidity data and stores them on the local storage server. A thread is started each time a new thermal image is stored on the local storage server, assuming live meteorological data are present. It first conducts a nearest-neighbor search to synchronize the data, pairing the thermal image taken with the milking data point closest in time within a 5-min tolerance window. Then, an algorithm based on a SegFormer segmentation neural network performs udder segmentation of the thermal image and extracts the temperature distribution of the area of interest, accounting for atmospheric temperature and humidity measured by the weather station. Lastly, it determines the maximum temperature within the segmented region and evaluates it against pre-defined threshold values along with the corresponding somatic cell count, providing real-time udder health indicators that support veterinarians in identifying potential subclinical mastitis. By comparing the timestamp of the thermal image with the milking robot’s RFID recognition system, the algorithm is able to correctly associate each thermal image with the animal that produced it, which can then be immediately identified and isolated in the event that problems or anomalies are detected in its temperature measurement.

### 2.6. Synchronization of Thermography with the Milking Robot

The synchronization between the thermography system and the milking robot is managed by a Python script that performs an automated data alignment process. Each time a new thermal image is uploaded to the FTP server, or at regular intervals, the script detects the file, reads the associated timestamp, and compares it with the milking records from the robot. These records provide a detailed report on each buffalo’s milking session, including the exact time and the animal’s ID. The script cross-references this data, linking each thermal image to the corresponding milking session, thus uniquely identifying the animal. This system enables continuous and automated monitoring of udder health, ensuring seamless integration of thermography into the milking routine without disrupting operations.

## 3. Core Contribution: A SegFormer Neural Network for Udder Segmentation and Thermal Feature Extraction

This section presents the core technical innovation of the proposed system: a transformer-based segmentation neural network (SegFormer), specifically trained to accurately segment the buffalo udder in thermal images acquired during milking. The model enables automatic extraction of the maximum udder surface temperature, corrected for environmental conditions, and supports real-time udder health monitoring. This AI module constitutes the foundation of the automated diagnostic approach developed in this work.

However, automatic and continuous data acquisition alone is not sufficient to provide meaningful information; it must be accompanied by appropriate analysis [12].

For example, using a very simple algorithm, it is possible to extract the maximum recorded temperature from each thermal image captured and use it as an index of inflammation and, therefore, likely mastitis. However, the thermal image captured by the infrared camera includes not only the target udder but also other areas close to it, such as the abdomen, the inner thigh, and elements of the milking robot.

Therefore, if the recorded maximum temperature does not coincide with a point on the udder, the result would be an error in measuring the maximum udder temperature. This could lead to a false diagnosis [13]. To overcome this problem, a region of interest (ROI) should be selected within the thermal image that coincides with the udder area, and temperature extraction and analysis should be limited to this ROI.

The provided camera tools already allow the definition of an ROI within the image of different shapes (square, circular, and elliptical) and sizes, from which the maximum temperature can be extracted. However, whatever ROI is chosen, it can never perfectly coincide with the udder area, which has a very irregular morphology, leading to a possible loss of measurement accuracy.

**Figure 9:**
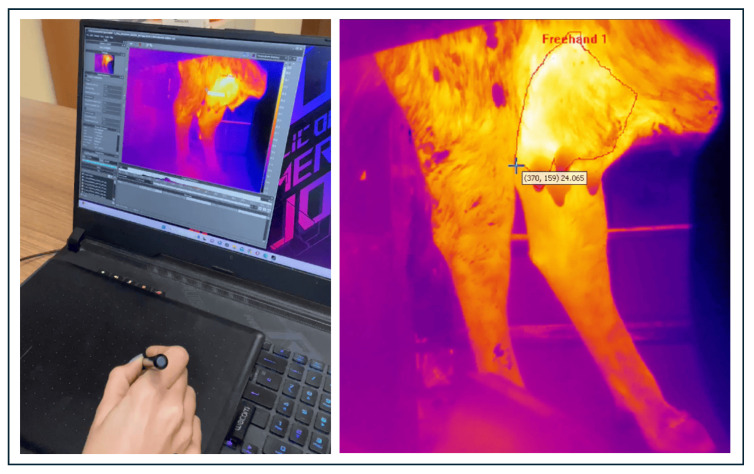
Example of hand-determination of the udder ROI.

The first solution explored by the authors was to exploit another possibility offered by the automatic image analysis tool of the thermal camera. Namely, defining the ROI on the thermal image by hand (freehand ROI). This operation consists of manually selecting a region of interest in an image, usually by drawing an irregular shape with a mouse or stylus on a tablet and was used to locate the breast (Figure 9).

However, the potential advantages of the freehand ROI technique can be quickly negated because the manual selection is slow and can become impractical for lots of complex images with many details. In particular, the Italian Mediterranean buffalo has a less standardized mammary morphology than cows. The udders are characterized by shorter teats, and the mammary quarters are less symmetrical and have a more irregular distribution [14].

Significant differences are also observed between individuals of the same species. The segmentation of the udder of a Mediterranean buffalo requires a high level of attention, which inevitably decreases over time due to the fatigue effect on the operator, who may be inclined to accidentally include or exclude important parts, further reducing accuracy.

Therefore, the solution to realize a real-time, large-scale mastitis diagnosis system based on infrared thermography lies in the integration of the acquisition system with a suitably trained segmentation neural network model along with a temperature extraction algorithm that, for each thermal image,

1. segments the udder;
2. extracts the maximum temperature from the segmented area accounting for meteorological factors;
3. evaluates the temporal relationship between the maximum udder temperature and other key biological parameters, providing real-time udder health indicators that assist veterinarians in assessing the risk of mastitis.

### 3.1. Architecture

The problem addressed by semantic segmentation is assigning labels (classes) to each pixel of an image. There are several ways to do it for neural networks, but the most commonly used is convolutional neural networks (CNNs). In recent years, though, because of the rise in popularity of transformers, they have also been massively used for semantic segmentation. Such vision transformers include ViT [15] and SETR [16]. The performances of these models are indeed outstanding, yet they face challenges when compared to more traditional approaches, such as the lack of multi-scale features and the fixation of positional encoding, which are unable to handle images with variable resolutions. SegFormer addresses these limitations by proposing a hierarchical transformer architecture.

SegFormer [17] is essentially a hierarchical vision transformer encoder that inherits the multi-scale feature extraction idea from CNNs. It first splits an input image into small 4×4 patches, which are then embedded by convolutional layers before being fed into the transformer-based SegFormer blocks, which guarantee overlapping between the patches. Then, as the input image passes through the different stages of the encoder, these patches are merged to form feature maps of lower resolutions (1/4,1/8,1/16,1/32).

The transformer-based Segformer blocks that characterize the encoder propose two key innovations: efficient self-attention and Mix-FFN (feed-forward network).

- Efficient Self-Attention: Standard self-attention mechanisms are computationally expensive. SegFormer addresses this limitation by introducing a reduction operation on key *K* and value *V* matrices before computing self-attention;
- Mix-FFN: Standard transformers employ fixed positional encodings, which are not suitable for varying image resolutions when compared to the training data. SegFormer addresses this limitation by using a depthwise convolution layer, allowing spatial information to be implicitly preserved.

The decoder shares the same architecture philosophy as the encoder by being intentionally simple, relying entirely on multi-layer perceptrons (MLP).

The encoder produces multi-scale feature maps $\left(F_{1},F_{2},F_{3},F_{4}\right)$ from the different stages of the encoder. The decoder aggregates these multi-scale features using MLP layers to normalize the channel dimensions and bilinear interpolation upsampling to concatenate them. Notably, the highest-resolution feature map bypasses upsampling, while others are progressively upsampled by factors of 2, 4, and 8, respectively.

These innovations, along with the idea of having patches with shared spatial (overlapping) information inspired by methods like the pyramid vision transformer (PVT) [18] and Swin transformer [19], allow SegFormer to be both an efficient and performative segmentation network.

**Figure 10:**
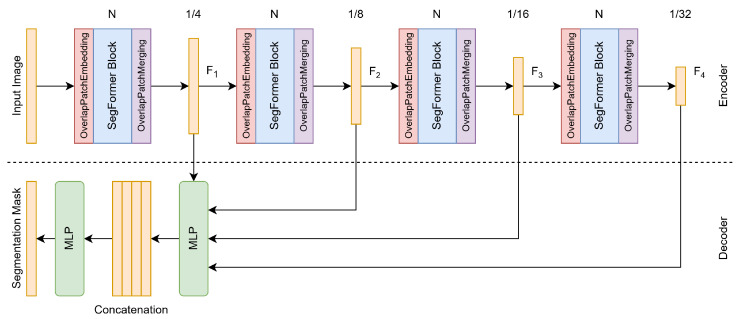
Depiction of the SegFormer architecture at a high level.

The overall architecture described above is depicted at a high level in Figure 10.

As with most models, the SegFormer model proposed by the authors is available in multiple configurations. Table 1 summarizes the key characteristics of different variants, where:

- Enc Depths represent the number of transformer layers in each encoder block;
- Enc Sizes represent the dimensionality of feature representations at different stages in the encoder;
- Dec Sizes represent the number of feature dimensions processed by the decoder;
- Params represent the total number of model parameters in millions, representing model complexity.

In this study, we employ the Segformer variant with the MiT-b5 backbone for our segmentation task.

### 3.2. Dataset and Preprocessing

The self-collected dataset used in this study comprised 2148 thermal images of buffalo udders, which were divided into three sets, namely training, validation, and test sets, in a 70/20/10 fashion.

It is important to note that the entire dataset, including the thermal images, was exclusively collected by the authors and is not derived from any publicly available source.

**Figure 11:**
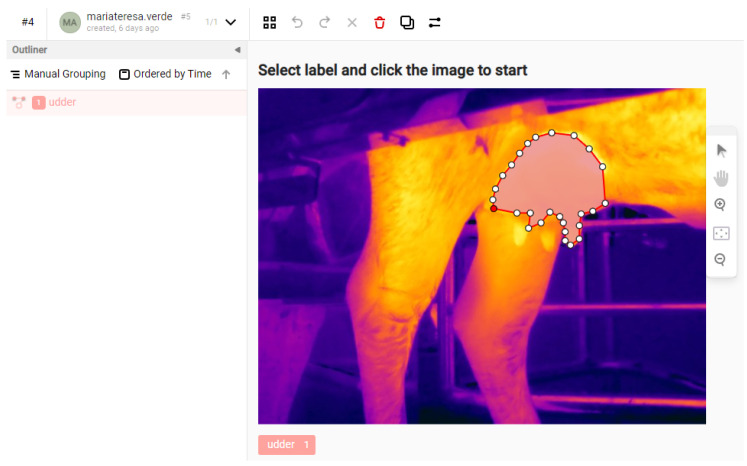
Ground truth obtained through experts labeling images in Label Studio.

The 2148 thermal images were manually annotated by two veterinarians with extensive experience in udder pathology in Italian Mediterranean buffalo. The annotation was carried out using the open-source software Label Studio, which is widely used in medical and veterinary image segmentation tasks and allows for the precise definition of segmentation polygons [20] (Figure 11). To ensure consistency and accuracy, a shared annotation protocol was established prior to labeling, including examples of correct segmentations, inclusion/exclusion criteria (e.g., visible teats and non-parenchymal regions), and specific guidelines for non-standard udder morphologies. Intra- and inter-observer consistency was assessed by having both experts annotate a randomly selected subset of images. In cases of significant discrepancy, joint review and consensus-based re-annotation were performed.

The thermal images were converted from the raw temperature matrices to a single-channel image using min–max normalization, reducing the input sizes for optimal performances. Moreover, several reversible image augmentation techniques, including flipping, rotation, and translation, were applied to the training dataset to enhance the model’s generalization capabilities.

No additional filtering or morphological smoothing was applied to the predicted masks, as they already exhibited good morphological consistency and accuracy.

### 3.3. Hyperparameter Optimization

We employed a Bayesian optimizer [21] to find the optimal hyperparameter configuration that maximizes the mean intersection-over-union (mIoU) score over the test set of the proposed SegFormer segmentation model. For this application, we used the Weights and Biases Sweeps [22] tool. The hyperparameter space used for the search included (i) batch size, (ii) learning rate, and (iii) loss function parameters, specifically a weight for the two of them and a smoothing factor for the soft binary cross entropy. The specifications of the hyperparameter space explored are reported in Table 2.

**Figure 12:**
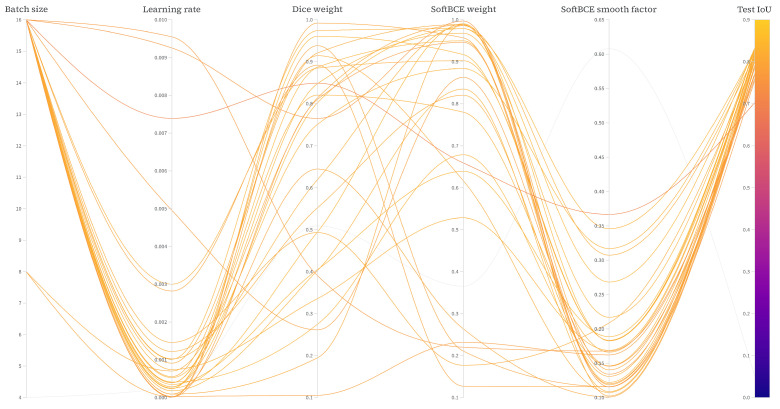
Comparison of the mIoU score over the test set for different configurations explored by the Bayesian optimizer.

Since Segformer is a transformer-based architecture, it is computationally expensive, especially when paired with the MiT-b5 backbone. For this reason, the optimization process was performed using just 33% of the training set, 30 epochs per configuration tested, the smallest SegFormer backbone available (MiT-b0), and a total count of 50 unique configurations. The best-performing hyperparameter configuration found, in terms of mIoU over the test set, is reported in Table 3, while all the other hyperparameter configurations tested, along with their test set mIoU, are depicted in Figure 12.

### 3.4. Training Process

We enhanced the SegFormer model’s shape and pixel-level segmentation accuracies by combining the following loss components, where each of them focused on a different aspect of the segmentation task.

- Soft Binary Cross Entropy: This focuses on pixel-level accuracy, where the segmentation problem is treated as a pixel-per-pixel classification problem. A smoothing factor addresses possible labelling errors/noise in the annotations [23].
- Binary Dice Loss: This focuses on the segmentation task’s shape-level goodness. It emphasizes the overall shape and overlap of predicted and actual segmentation masks, ensuring that the model captures the proper udder structure [24].

These loss components are combined in one loss function as the weighted sum of their components. We also employed an AdamW optimizer and a learning rate scheduler, which decreases the learning rate by a 0.5 factor if there is no improvement in validation loss within 10 epochs. The model was finally trained for up to 200 epochs, with an early stopping callback set after 50 epochs without any improvement. The weights for the backbone were not randomly initialized; instead, an official checkpoint from training on the ImageNet dataset [25] was used.

### 3.5. Model Performance

The mIoU measures the overlap of the predicted and actual segmentation regions against their union, while the F1-score measures the balance between precision and recall at a pixel level.

The best performance of the model was reached at epoch 48 for a training procedure that lasted 98 epochs, for which the mean intersection over union (mIoU) and F1-score over the test set were 0.827 and 0.899, respectively.

These scores hint at high accuracy in segmenting udder regions with minimal false positives and negatives.

**Figure 13:**
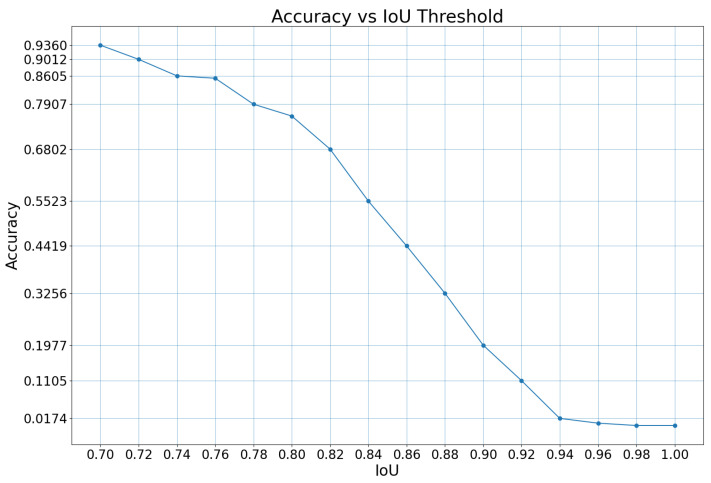
Plot of detection accuracy at varying IoU thresholds.

To evaluate the model’s ability to detect an udder region within a thermal image automatically, we computed an accuracy metric at an image level as the percentage of correct udder region detection within the test set. An udder detection is considered correct when the IoU between the predicted and actual segmentation of the udder region is higher than a threshold. We consider this threshold acceptable for our task, starting from 0.80, for which the model reaches 77.9% accuracy (Figure 13).

### 3.6. Temperature Extraction Accounting for Meteorological Factors

When the temperature measurement algorithm extracts the temperature distribution of the udder region of the thermal image, it accounts for both atmospheric temperature and humidity. Thermal imaging aims to estimate the surface temperatures of objects, which in this case is the udder skin surface temperature (USST), by measuring their emitted infrared radiation. Although this holds true in theory, in practice, the measured radiation is affected by the object itself, the atmosphere and the interaction between the two. Specifically, the atmosphere emits an infrared radiance, and the object reflects part of this radiation. In the long-wave infrared range (LWIR), water vapor is the principal absorber, making humidity and atmospheric temperature critical factors. The measured radiation is not only affected by the object’s (udder’s) temperature but also by parameters like (i) object emissivity ϵ, (ii) atmospheric temperature $T_{atm}$, (iii) relative humidity, and (iv) the object’s distance from the thermal camera *D*. Atmospheric temperature and relative humidity determine atmospheric transmittance τ and self-emission (1−τ). The atmospheric transmittance τ equation describes how infrared radiation is attenuated over the distance from thermal camera *D*. The equation is provided as follows:

(1) $$\tau =X\;\exp\;\left(-\sqrt{D}\;\left(A_{1}+B_{1}\;\sqrt{p_{H_{2}O}}\right)\right)+\left(1-X\right)\;\exp\;\left(-\sqrt{D}\;\left(A_{2}+B_{2}\;\sqrt{p_{H_{2}O}}\right)\right),$$

where $p_{H_{2}O}$ is the water vapor partial pressure (derived from relative humidity and atmospheric temperature) and *X*, $A_{1}$, $A_{2}$, $B_{1}$, and $B_{2}$ are camera calibration parameters.

The total radiance $W_{tot}$ can be now written as follows:

(2) $$W_{tot}=\epsilon \tau W_{obj}+\left(1-\epsilon \right)\tau W_{refl}+\left(1-\tau \right)W_{atm},$$

where $W_{obj}$, $W_{refl}$, and $W_{atm}$ are blackbody-like radiances corresponding to the object temperature, the ambient reflected temperature, and the atmospheric temperature, respectively. Assuming that the camera signal values *U* are proportional to the corresponding radiances by a factor *C* such that U=CW and that the camera’s internal calibration sets this factor, it is now possible to estimate $U_{obj}$ from $W_{obj}$ from Equation (2) [26].

The meteorological corrections adopted (based on continuous measurements of temperature and humidity through a weather station and the application of a calibrated physical model) were designed to be independent of the specific context.

To enhance temperature accuracy, the atmospheric temperature and relative humidity, recorded every 20 min by the weather station, are associated with each thermal image using a nearest-neighbor search within a ±5-min time window. These values feed into a physical correction model based on transmittance and radiance equations, designed to compensate for atmospheric absorption effects, particularly those due to water vapor. This step allows a more precise estimation of the actual udder skin surface temperature (USST), thereby improving diagnostic reliability under real-world farm conditions.

### 3.7. Maximum Temperature Measurement Results

We investigated the effectiveness of such an automatic measurement system based on the AI segmentation model. According to the predicted and actual udder regions within the test set of thermal images, we extracted the temperature distributions from both of them utilizing the official FLIR SDK, the software from the thermal camera manufacturer. We applied a thermal correction process based on meteorological factors, whose logic is explained in Section 3.6, to ensure accurate temperature readings, adjusting each image for emissivity, distance, ambient temperature, and relative humidity. The emissivity and distance were set to 0.98 [27] and 1.0, respectively, while the atmospheric components were set according to the capture timestamp of each thermal image.

**Figure 14:**
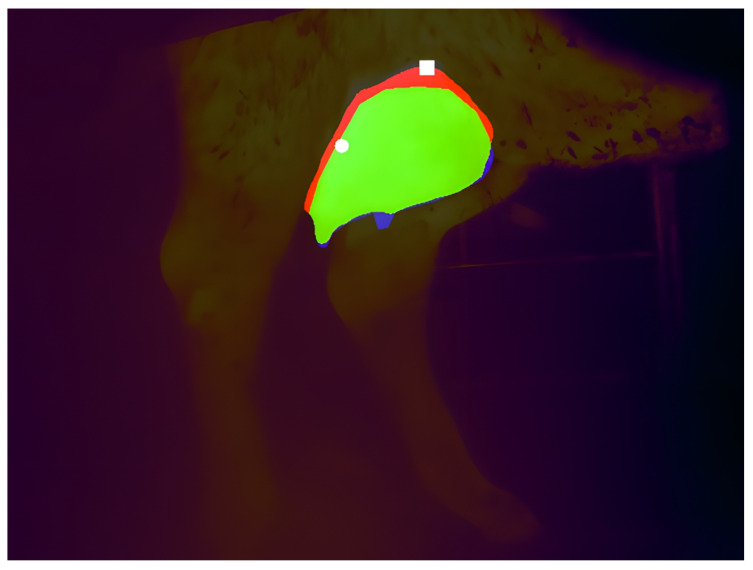
An example comparing the predicted segmentation mask by the AI model to the actual mask detected by the veterinaries: correctly segmented areas are green, incorrectly unsegmented areas are blue, and incorrectly segmented areas are red; a white square indicates the maximum temperature location in the predicted mask, while a white circle marks the corresponding location in the actual mask.

We then compared the maximum temperatures located over the udder regions detected by the AI model to the ones detected by the veterinarians. For these temperature pairs, only 6.5% showed discrepancies. An example of such a discrepancy is illustrated in Figure 14. To quantify such discrepancies, we provide the descriptive statistics of the absolute differences of the maximum temperature pairs in Table 4. For such pairs, the mean absolute difference was 0.159 °C.

**Figure 15:**
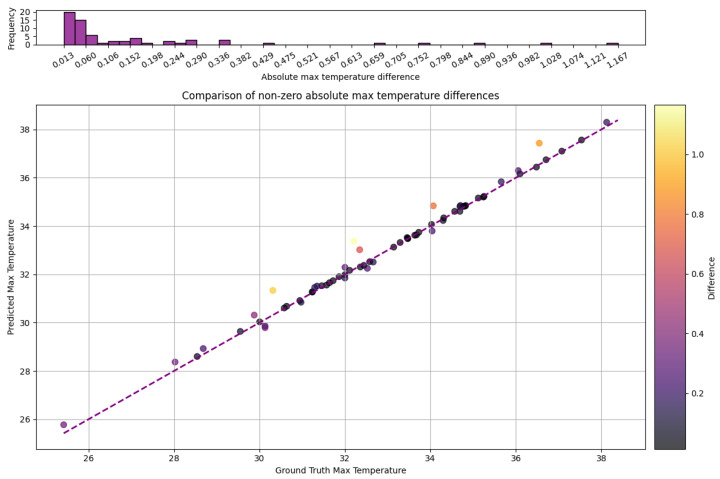
Comparison of udder maximum temperatures detected by our system and actual ones for cases where the temperatures do not coincide.

To better understand the temperature pair discrepancies, we plotted these pairs one against the other (Figure 15). As expected from the descriptive statistics in Table 4, most pairs that did not match are located near the bisector line with an order of magnitude of $10^{-2}$ according to the median.

In the application, the most common segmentation model error involves the failure to detect a single teat or a marginal portion of the mammary area. This type of error primarily leads to a local underestimation of the maximum temperature of the mammary region, a parameter that serves as the key indicator in our system. However, such errors occur infrequently (only in 6.5% of cases), and the differences in maximum temperature between predicted masks and expert-annotated ground truth masks remain limited. Therefore, given the distribution of errors, the practical impact on clinical decision-making is minimal.

## 4. First Tests on the Developed System for Monitoring Diseases

The study was conducted at a commercial buffalo farm in Cancello e Arnone, in Caserta, southern Italy (41.0624° N and 14.0378° E), with about 1000 animals. The trial was conducted in an experimental barn equipped with a DeLaval VMS300 milking robot. Initially, 80 primiparous animals were introduced into the barn to allow them to adapt more quickly to the robot. Despite the precautions taken, 17 animals failed to adapt to robotic milking and were removed from the group.

The exclusion of 17 buffalo from the study was based on clearly defined behavioral, physiological, and morphological criteria. Specifically, an animal was considered “non-adapted” if the following were true:

1. It persistently refused to voluntarily enter the milking robot or showed signs of stress or agitation (e.g., restlessness, vocalizations, and kicking);
2. It exhibited a marked and sustained reduction in milk production;
3. It presented unfavorable morphological characteristics for adaptation to the AMS, such as body height or teat positioning that impeded the proper functioning of the robotic arm.

Studies on Mediterranean buffalo have shown that teat canal length, canal diameter, and the relative positioning of teats significantly affect “milkability”, the ability to be milked efficiently, in automatic milking systems. These exclusion criteria were adopted from the initial adaptation phase to safeguard animal welfare and ensure the proper functioning of the robot, avoiding results influenced by morphological anomalies or stress factors unrelated to the milking system. Therefore, it is true that the analyzed group, including only adaptable individuals, reduced the representativeness of the sample relative to the overall buffalo population. However, the results remain valid for farms that already use or plan to adopt AMS technology, as they reflect real-world operating conditions for suitable animals. Future studies should also include non-adaptable buffalo in order to improve the robotic system design and make it more inclusive of animals with diverse body conformations.

The buffalo were housed in free stalls with approximately 18 m^2^ of space per head and approximately 80 cm of trough per animal.

The feeding system administered a total mixed ration (TMR), distributed twice daily. Buffalo that could be milked entered the holding area to the side of the AMS and moved independently to the milking station. Otherwise, they were diverted to the feeding area. The AMS used a standard configuration similar to that used for dairy cows, with the following operating parameters: vacuum at 42 kPa, 60 cycles/min, and a pulse ratio of 60%. The amount of concentrate fed to each buffalo at the milking station varied from 0.5 to 3.0 kg/day, depending on daily milk production. This adaptive system made it possible to optimize the feeding of the buffalo by providing them with amounts of concentrate proportional to their actual productivity, reducing waste, and increasing the efficiency of the production process.

For a data collection campaign counting 728 milking instances run over May 2024 and June 2024, we performed a statistical analysis to evaluate the potential for the proposed automatic measurement system.

The data collected included thermal camera images of buffalo udders, weather station meteorological data, and automated milking machine biological data on milk properties. More specifically, the biological data included online cell count (OCC) values and an estimate for somatic cell count (SCC) values, which are widely used as markers for udder mastitis infection. Elevated SCC levels are considered to be correlated to the presence of mastitis or other inflammatory diseases [28].

This analysis aimed to assess if higher OCC values were associated with higher maximum udder temperatures recorded by our system, since if the temperature measurements were correct, this trend would be present [28]. By conducting such an analysis, we wanted to explore the possibility of using such an automatic system to monitor udder health in real-time with the non-invasive approach provided by thermal imaging.

The 730 milking instances were divided into two groups according to the udder health of the buffalo that produced such milk. Of these milkings, 686 were from a healthy buffalo, while 44 were from a mastitis-affected buffalo. Milkings were classified as from a mastitis-affected buffalo if the OCC in raw milk for that milking session exceeded 400,000 cells/mL [28].

We investigated if there was a statistically significant temperature shift between the two milking groups; one originated from healthy buffalo, and the other from mastitis-affected ones. The mean maximum udder temperature for healthy buffalo was 33.48 °C ± 1.84 °C, while for the others, it was 34.19 °C ± 1.39 °C, resulting in a difference of 0.71 °C on average.

This absolute difference aligns with the existing literature, where the maximum udder temperature for mastitis-affected buffalo is higher due to the inflammatory response associated with the disease [29]. Specifically, although most of it refers to cows rather than buffalo, scientific investigations report that the udder surface temperature of subclinical mastitis and clinical mastitis-affected mammal quarters are, respectively, 0.72 °C and 1.05 °C higher, on average, than the udder surface temperature of unaffected quarters [30]. In this analysis, our automated system captured this phenomenon, highlighting its potential to detect early signs of health issues in buffalo udders automatically and non-invasively.

**Figure 16:**
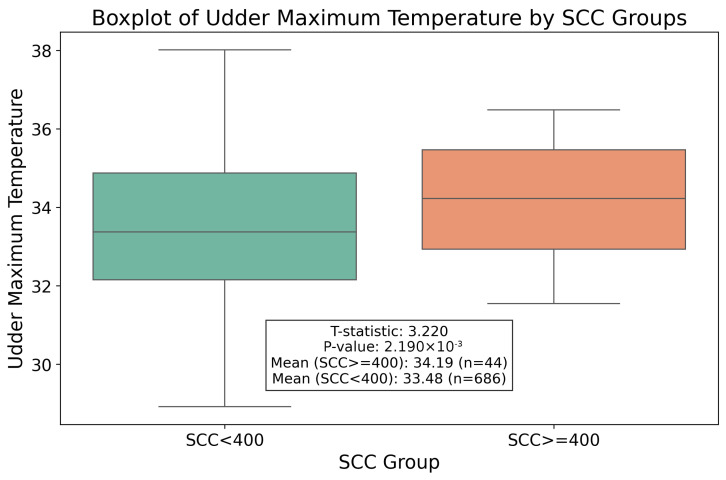
Boxplot of the measured udder maximum temperature for the two OCC groups.

To confirm the significance of this temperature difference, we conducted a *t*-test. At first, we conducted Levene’s test for variance equality, which yielded a *p*-value of 0.0415, indicating that variances were not equal. Then, we conducted Welch’s *t*-test, which did not assume equal variances. It yielded a *p*-value of 0.0022, as illustrated in Figure 16.

The *p*-value, which is lower than the standard threshold of 0.05, indicates that the temperature shift between the mean temperatures of the two groups is statistically significant. This finding supports the initial hypothesis that the maximum udder temperatures measured by our automatic system are correlated with the udder health status of buffalo, since higher values of OCC are associated with higher maximum udder temperatures.

### Potential Impact of NSAID Application

As an additional potential application of the proposed automatic udder temperature system, we monitored the effects of a non-steroidal anti-inflammatory drug (NSAID) treatment on the udder health of six buffalo diagnosed with mastitis, a condition confirmed by our veterinary expert within the innovative farm.

**Figure 17:**
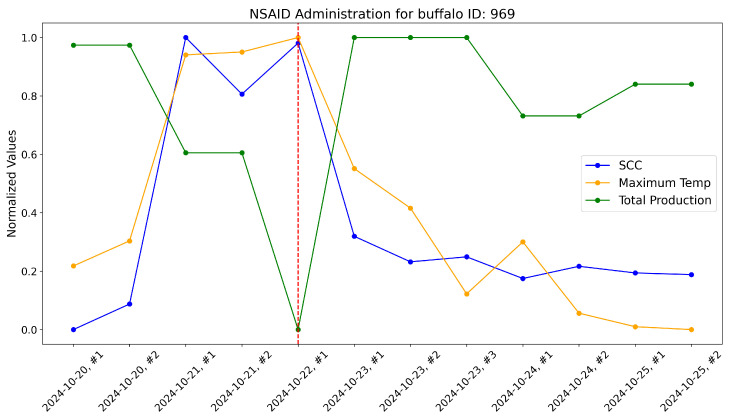
Raw data plot for buffalo with ID 969.

**Figure 18:**
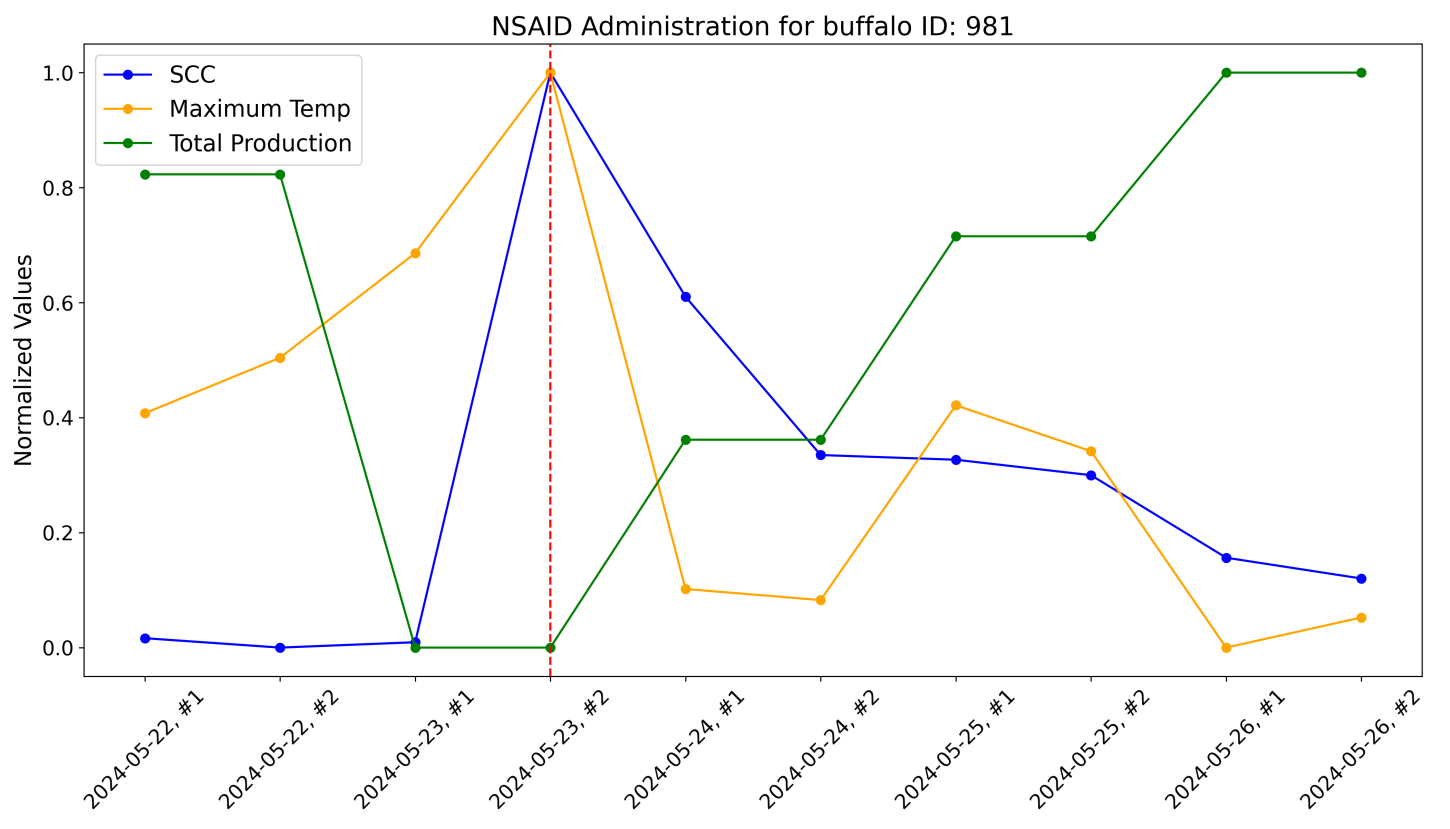
Raw data plot for buffalo with ID 981.

**Figure 19:**
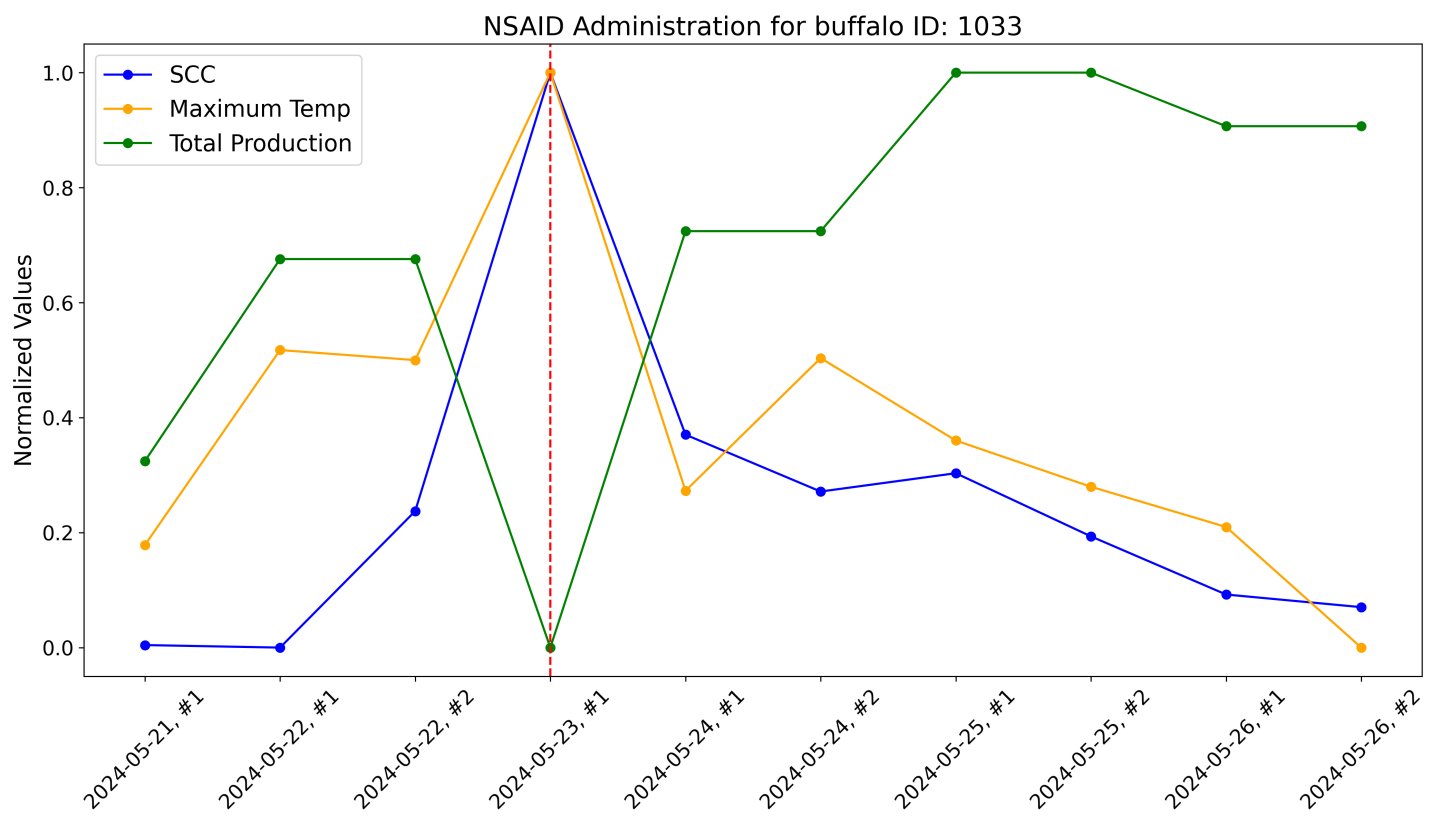
Raw data plot for buffalo with ID 1033.

**Figure 20:**
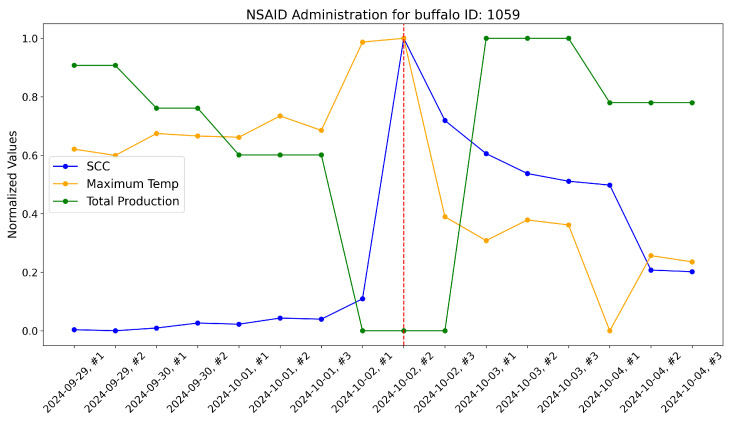
Raw data plot for buffalo with ID 1059.

**Figure 21:**
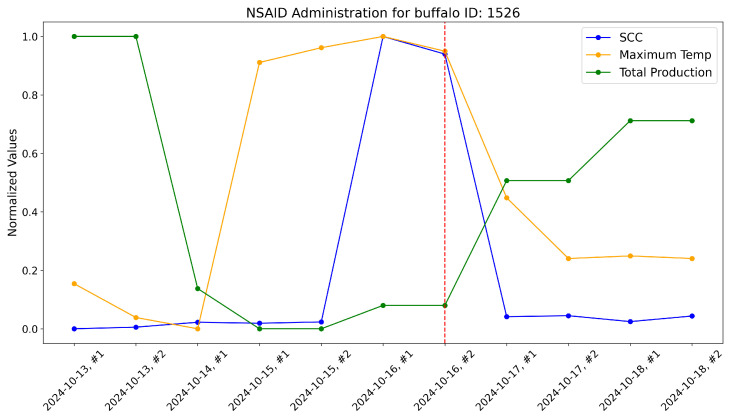
Raw data plot for buffalo with ID 1526.

**Figure 22:**
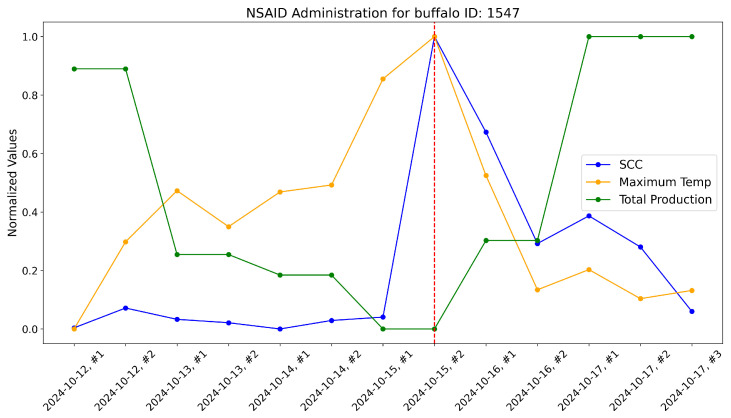
Raw data plot for buffalo with ID 1547.

According to the veterinarians, these buffalo exhibited behaviors typically associated with subclinical mastitis. For these, the maximum udder temperature monitored by our system increased well before a significant rise in the somatic cell count, which usually marks the onset of mastitis infection. These trends were evident when comparing the variations in these variables over time within the same plot. As a reference, the time-aware variations in maximum udder temperature, somatic cell count, and milk production (normalized) are shown for these specific animals in Figure 17, Figure 18, Figure 19, Figure 20, Figure 21 and Figure 22.

When the increase in the maximum temperature of the udder was considered significant by the expert veterinarians who monitored the animals using the automated system, they administered non-steroidal anti-inflammatory drugs (NSAIDs) instead of more commonly used antibiotics, which are typically employed in treating mastitis.

This approach yielded a positive effect, as shown by the graphs, with a rapid reduction in temperature, a decrease in the somatic cell count, and the recovery of adequate milk production levels following treatment. In practice, timely mastitis diagnosis through thermographic inspection allowed by our system led to effective treatment with NSAIDs because the condition was still in its early stages. This resulted in an overall milk quality and quantity benefit compared to traditional diagnosis and treatment methods. Furthermore, it is well known that avoiding antibiotics helps counteract phenomena like antibiotic resistance, which threatens health.

Early treatment with non-steroidal anti-inflammatory drugs offers several advantages. In terms of quality, it prevents macroscopic alterations in milk, ensuring a high overall standard. From an economic perspective, it helps avoid interruptions in sales. Finally, regarding animal welfare, timely intervention limits the spread of inflammatory signs and promotes a rapid recovery of the animal. For improved clarity, the data gathered for each animal are presented in the Table 5, Table 6, Table 7, Table 8, Table 9 and Table 10.

The raw data, along with plots, of the potential impact of the measurement system on NSAID application are reported here. Notice that dashed lines represent when NSAIDs have been administered to the buffalo.

The transition from a monitoring system to a fully autonomous diagnostic tool will require several critical steps. In particular, it will be necessary to

1. increase the sample size and validate the results on independent populations;
2. define reliable decision thresholds to distinguish between pathological and physiological states;
3. address regulatory requirements for clinical use;
4. investigate the integration of the system into existing veterinary workflows, ensuring usability, result interpretability, and interoperability with other management tools.

These developments will be the focus of future studies, but the preliminary results obtained represent an important step toward automated diagnostics.

## 5. Conclusions

This study presents a promising approach for the early detection of subclinical mastitis in Mediterranean buffalo using an AI-driven thermographic system integrated with automated milking robots. The system successfully addresses key challenges in buffalo mastitis detection, such as consistency in udder positioning and temperature variations due to environmental factors. Preliminary testing in a farm environment yielded positive results, demonstrating the system’s potential for real-time, automated monitoring.

The method’s effectiveness was evaluated using two primary metrics: the mean intersection over union (mIoU) for udder segmentation accuracy and the correlation between the maximum udder temperature and somatic cell count (SCC). The developed Segformer model achieved an mIoU of 0.827 and an F1-score of 0.899, indicating high accuracy in segmenting udder regions within thermal images. In addition, temperature monitoring was found to correlate strongly with SCC levels, with a statistically significant temperature difference of 0.71 °C between healthy buffalo and those with high SCC levels (*p*-value = 0.00219). This correlation underscores the system’s ability to detect subclinical inflammation through temperature anomalies.

In addition, the system demonstrated high practical reliability: 77.9% accuracy in detecting udder regions, with a low margin of error of 0.159 °C in temperature measurements. These results suggest that the method may provide a viable, scalable alternative to traditional diagnostic tools, offering a faster, non-invasive, and cost-effective means of monitoring buffalo udder health in large herds. Future development will focus on optimizing data integration from heterogeneous sources to improve detection robustness, characterize the temperature variations over time, and refine the AI algorithms for improved adaptability to varying farm conditions.

Moreover, future system upgrades will aim to implement a bilateral or mobile thermal imaging setup, enabling full udder visualization. This evolution is expected to enhance segmentation accuracy, improve the identification of localized inflammation, and increase the robustness of predictive models, supporting broader applicability across diverse farm settings.

## Figures and Tables

**Table 1:** SegFormer model variants.

Variant	Enc Depths	Enc Sizes	Dec Size	Params
MiT-b0	[2, 2, 2, 2]	[32, 64, 160, 256]	256	3.7
MiT-b1	[2, 2, 2, 2]	[64, 128, 320, 512]	256	14.0
MiT-b2	[3, 4, 6, 3]	[64, 128, 320, 512]	768	25.4
MiT-b3	[3, 4, 18, 3]	[64, 128, 320, 512]	768	45.2
MiT-b4	[3, 8, 27, 3]	[64, 128, 320, 512]	768	62.6
MiT-b5	[3, 6, 40, 3]	[64, 128, 320, 512]	768	82.0

**Table 2:** Hyperparameter space for Bayesian optimization.

Hyperparameter	Distribution	Range/Values
Batch Size	Categorical	{4, 8, 16}
Dice Weight	Uniform	[0.1, 1]
Learning Rate	Log-Uniform	[$10^{-5}$, $10^{-2}$]
SoftBCE Smooth Factor	Uniform	[0.1, 0.9]
SoftBCE Weight	Uniform	[0.1, 1]

**Table 3:** Optimal hyperparameter settings.

Hyperparameter	Optimal Value
Batch Size	8
Dice Weight	0.55
Learning Rate	$2\times 10^{-4}$
SoftBCE Smooth Factor	0.29
SoftBCE Weight	0.48

**Table 4:** Maximum udder temperature differences descriptive statistics.

Mean	Std. Dev.	Min	25th Pctl.	Median	75th Pctl.	Max
0.159	0.242	0.013	0.028	0.055	0.172	1.167

**Table 5:** Raw data gathered for buffalo with ID 969.

Animal	Milking	Milk	SCC	Udder Maximum
**ID**	**Date and Number**	**Yield**		**Temperature (°C)**
969	20 October 2024 #1	14.71	163	32.47
969	20 October 2024 #2	14.71	209	32.73
969	21 October 2024 #1	10.88	689	34.66
969	21 October 2024 #2	10.88	587	34.69
969	22 October 2024 #1	4.59	679	34.84
969	23 October 2024 #1	14.98	331	33.48
969	23 October 2024 #2	14.98	285	33.07
969	23 October 2024 #3	14.98	294	32.18
969	24 October 2024 #1	12.19	255	32.72
969	24 October 2024 #2	12.19	277	31.98
969	25 October 2024 #1	13.32	265	31.84
969	25 October 2024 #2	13.32	262	31.81

**Table 6:** Raw data gathered for buffalo with ID 981.

Animal	Milking	Milk	SCC	Udder Maximum
**ID**	**Date and Number**	**Yield**		**Temperature (°C)**
981	22 May 2024 #1	6.63	51	32.66
981	22 May 2024 #2	6.63	37	33.01
981	23 May 2024 #1	5.56	45	33.67
981	23 May 2024 #2	5.56	894	34.81
981	24 May 2024 #1	6.03	560	31.55
981	24 May 2024 #2	6.03	324	31.48
981	25 May 2024 #1	6.49	317	32.71
981	25 May 2024 #2	6.49	294	32.42
981	26 May 2024 #1	6.86	171	31.18
981	26 May 2024 #2	6.86	140	31.37

**Table 7:** Raw data gathered for buffalo with ID 1033.

Animal	Milking	Milk	SCC	Udder Maximum
**ID**	**Date and Number**	**Yield**		**Temperature (°C)**
1033	21 May 2024 #1	6.38	42	31.89
1033	22 May 2024 #1	10.0	38	32.86
1033	22 May 2024 #2	10.0	254	32.81
1033	23 May 2024 #1	3.04	948	34.24
1033	24 May 2024 #1	10.5	375	32.16
1033	24 May 2024 #2	10.5	285	32.82
1033	25 May 2024 #1	13.34	314	32.41
1033	25 May 2024 #2	13.34	214	32.18
1033	26 May 2024 #1	12.38	122	31.98
1033	26 May 2024 #2	12.38	102	31.38

**Table 8:** Raw data gathered for buffalo with ID 1059.

Animal	Milking	Milk	SCC	Udder Maximum
**ID**	**Date and Number**	**Yield**		**Temperature (°C)**
1059	29 September 2024 #1	14.77	6	33.5
1059	29 September 2024 #2	14.77	4	33.4
1059	30 September 2024 #1	13.68	9	33.75
1059	30 September 2024 #2	13.68	18	33.71
1059	01 October 2024 #1	12.49	16	33.69
1059	01 October 2024 #2	12.49	27	34.03
1059	01 October 2024 #3	12.49	25	33.8
1059	02 October 2024 #1	8.01	62	35.21
1059	02 October 2024 #2	8.01	534	35.27
1059	02 October 2024 #3	8.01	385	32.42
1059	03 October 2024 #1	15.46	325	32.04
1059	03 October 2024 #2	15.46	289	32.37
1059	03 October 2024 #3	15.46	275	32.29
1059	04 October 2024 #1	13.82	268	30.6
1059	04 October 2024 #2	13.82	114	31.8
1059	04 October 2024 #3	13.82	111	31.7

**Table 9:** Raw data gathered for buffalo with ID 1526.

Animal	Milking	Milk	SCC	Udder Maximum
**ID**	**Date and Number**	**Yield**		**Temperature (°C)**
1526	13 October 2024 #1	13.46	20	31.74
1526	13 October 2024 #2	13.46	25	31.35
1526	14 October 2024 #1	7.32	40	31.22
1526	15 October 2024 #1	6.34	37	34.29
1526	15 October 2024 #2	6.34	41	34.46
1526	16 October 2024 #1	6.91	914	34.59
1526	16 October 2024 #2	6.91	860	34.42
1526	17 October 2024 #1	9.95	57	32.73
1526	17 October 2024 #2	9.95	60	32.03
1526	18 October 2024 #1	11.41	42	32.06
1526	18 October 2024 #2	11.41	59	32.03

**Table 10:** Raw data gathered for buffalo with ID 1547.

Animal	Milking	Milk	SCC	Udder Maximum
**ID**	**Date and Number**	**Yield**		**Temperature (°C)**
1547	12 October 2024 #1	11.49	42	32.08
1547	12 October 2024 #2	11.49	77	33.46
1547	13 October 2024 #1	8.32	57	34.27
1547	13 October 2024 #2	8.32	51	33.70
1547	14 October 2024 #1	7.97	40	34.25
1547	14 October 2024 #2	7.97	55	34.36
1547	15 October 2024 #1	7.05	61	36.04
1547	15 October 2024 #2	7.05	557	36.71
1547	16 October 2024 #1	8.56	388	34.51
1547	16 October 2024 #2	8.56	191	32.70
1547	17 October 2024 #1	12.04	240	33.02
1547	17 October 2024 #2	12.04	185	32.56
1547	17 October 2024 #3	12.04	71	32.69

## Data Availability

The datasets used in this study are not publicly available as they are proprietary and were generated by the authors in the context of the current research. However, they can be made available upon reasonable request to researchers, universities, or research institutions for scientific purposes.
